# Decomposition–Coordination of Double-Layer MPC for Constrained Systems

**DOI:** 10.3390/e25010017

**Published:** 2022-12-22

**Authors:** Hongrui Wang, Pengbin Zhang, Zhijia Yang, Tao Zou

**Affiliations:** 1 State Key Laboratory of Robotics, Shenyang Institute of Automation, Chinese Academy of Sciences, Shenyang 110016, China; 2 Key Laboratory of Networked Control Systems, Chinese Academy of Sciences, Shenyang 110016, China; 3 Institutes for Robotics and Intelligent Manufacturing, Chinese Academy of Sciences, Shenyang 110169, China; 4 University of Chinese Academy of Sciences, Beijing 100049, China; 5 School of Mechanical and Electrical Engineering, Guangzhou University, Guangzhou 510006, China

**Keywords:** double-layer, dual decomposition, model predictive control

## Abstract

Large-scale industrial processes usually adopt centralized control and optimization methods. However, with the growth of the scale of industrial processes leading to increasing computational complexity, the online optimization capability of the double-layer model predictive control algorithm is challenged, exacerbating the difficulty of the widespread implementation of this algorithm in the industry. This paper proposes a distributed double-layer model predictive control algorithm based on dual decomposition for multivariate constrained systems to reduce the computational complexity of process control. Firstly, to solve the problem that the original dual decomposition method does not apply to constrained systems, two improved dual decomposition model prediction control methods are proposed: the dual decomposition method based on the quadratic programming in the subsystem and the dual decomposition method based on constraint zones, respectively. It is proved that the latter will certainly converge to the constraint boundaries with appropriate convergence factors for the controlled variables. The online optimization ability of the proposed two methods is compared in discussion and simulation, concluding that the dual decomposition method based on the constraint zones exhibits superior online optimization ability. Further, a distributed double-layer model predictive control algorithm with dual decomposition based on constraint zones is proposed. Different from the objective function of the original dual decomposition model predictive control, the proposed algorithm’s dynamic control-layer objective function simultaneously tracks the steady-state optimization values of the controlled and manipulated variables, giving the optimal solution formulation of the optimization problem consisting of this objective function and the constraints. The algorithm proposed in this paper achieves the control goals while significantly reducing the computational complexity and has research significance for promoting the industrial implementation of double-layer model predictive control.

## 1. Introduction

Modern industrial processes are characterized by large-scale components, extensive spatial structure, and strong sub-unit coupling [1]. The hierarchical optimization and control structure is often used for high-dimensional complex systems. It contains a planning and scheduling layer, real-time optimization (RTO) layer, advanced process control (APC) layer, and a regular control layer [2,3]. Model predictive control (MPC) is a type of APC algorithm that deals with multi-input multi-output constraint systems. The successful implementation of MPC can generate considerable revenues for companies, but its practical application is not universal for smaller companies with insufficient capacities. Due to the time scale between layers being different, disturbances entering into the process during any control period will result in a shift in the static operating point, and dynamic control cannot work on the optimal point, thus affecting economic efficiency. The optimal results of the RTO layer may not be suitable for current operating conditions. A two-stage MPC structure is commonly used in the industry [4,5,6,7], so many scholars proposed double-layer model predictive control (DLMPC). The steady-state optimization (SSO) layer of the DLMPC is able to adjust constraints and optimize steady-state operating points based on the steady-state model, while the dynamic control layer accomplishes the tracking of set values based on the dynamic control (DC) model [8,9,10,11].

The premise of implementing hierarchical optimization and control structure is based on a decentralized control system, realizing the coordination optimization among subsystems with control algorithms. Compared to centralized control systems, decentralized control systems can reduce computational complexity. Tamás Keviczky et al., facing a class dynamic decoupling systems’ optimal control problem, designed a decentralized rolling horizon control (RHC) scheme, describing the coupling between systems through diagrams, which decomposes centralized RHC controllers into some small RHC controllers to reduce the complexity of the problem and analyzes the sufficient stability conditions for the proposed solution [12]. For extensive dynamic processes with input constraints, Alessandro Alessio proposed decentralized model predictive control that approximated the global model by decomposing it into several smaller models for local predictions, giving sufficient conditions for asymptotic closed-loop stability in the absence of intermittent communication of measurement data [13]. Focusing on the lack of intermittent measurement data communication between decentralized model prediction controllers, Davide Barcelli gave sufficient criteria for the asymptotic tracking of output setpoints and rejection of constant measurement disturbances, and proposed a decentralized model predictive control method for extensive process setpoint tracking [14].

Although powerful algorithms quantify the interactions of decentralized control loops, decentralized control systems still lack the coupling between multiple variables [15]. In contrast to decentralized control systems, distributed control methods enable controllers to communicate with each other to cooperate while operating individually. Each controller considers the dynamic interactions among systems [16]. Aiming at the internet multi-objective optimal problem, Andrea Camisa proposed a distributed model predictive control (DMPC) calculation scheme of negotiation among agents based on cooperative game theory [17]. M. Francisco introduced a new fuzzy inference system into the cooperative game algorithm to propose multi-agent fuzzy negotiation distributed model predictive control considering economic criteria and process constraints on the negotiation process of the agents [18]. For the control of large urban traffic networks, Zhao Zhou et al. proposed a two-level hierarchical control framework, which uses the MPC method, with the upper level solving higher-level optimization problems for traffic-demand balance and the lower level adopting distributed control schemes within each sub-network to reduce the computational complexity [19]. A negotiation strategy among agents was designed using fuzzy rules under a hierarchical DMPC control architecture. The agents negotiate in pairs at the lower layers based on coupling and communication networks, with the lower-level negotiation process avoiding a portfolio explosion [20]. Under non-iterative, non-cooperative MPC algorithms, Marcello Farina proposed a DMPC algorithm that requires only partially connected communication networks and structural information [21]. In terms of non-cooperative strategies, Haimin Hu proposed new distributed iterative learning model predictive control that uses the local states and input trajectories of the previous iteration to construct time-varying safety sets and terminal cost functions [22]. For multi-area interconnected power systems, an algorithm based on Laguerre functions for DMPC containing the game-theoretic Nash equilibrium was proposed, with each regional DMPC controller coordinating with other controllers to find Nash equilibrium points [23].

Many scholars have proposed distributed or decentralized MPC algorithms based on dual decomposition. Joseph J. Yame et al. proposed a new DMPC containing both decomposition subsystem and integrated coordination sub-controller stages [24]. Takumi Namba et al. proposed a dual decomposition DMPC and applied it to a microgrid with the large-scale introduction of PV power [25]. Yuji Wakasa et al. proposed a decentralized model predictive control algorithm based on dual decomposition, which enables the decentralized control approach to solve the original optimization problem accurately using iterations [26]. Xi et al. proposed a decomposed–coordinated model predictive control (DCMPC) algorithm based on the theory of dual decomposition, but the algorithm is oriented towards DMC with only equation constraints without considering the case with constraints [2]. The authors used hierarchical distributed predictive control as a key search term to review the relevant literature [27,28,29,30,31]. At present, there are few research results on distributed control algorithms in the DLMPC structure. Yang Kai et al. proposed an integrated algorithm for real-time optimization and distributed control, with an overall economic optimization model for the upper layer and a distributed dynamic control structure for the lower layer [32]. Shi et al. proposed a distributed two-layer structure strategy for large-scale systems, with an online adaptive constraint adjustment scheme for the upper layer considering the possible constraints and priority order in the process. Based on the Pareto optimal algorithm, the lower layer proposed a new cooperative distributed dynamic matrix control based on a Jacobi-type iterative cooperation approach to achieve a globally optimal solution [33].

Centralized control optimization is often employed for industrial process control. However, the high computational effort of centralized control optimization challenges the MPC’s online optimization capabilities, exacerbating the difficulty of the widespread implementation of DLMPC algorithms in the industry. Reducing the more considerable computational complexity with a bit of sacrifice of control performance under satisfying the control goals is of greater significance in promoting the popularization of the industrial implementation of DLMPC algorithms. To reduce the computational complexity of industrial process control, research in this paper is oriented towards constrained multivariable distributed control systems. Based on decomposition–coordination MPC, we propose two strategies for adding constraints, and based on one of them we propose decomposition–coordination of DLMPC for constrained systems, with the following main contributions: Firstly, two methods are proposed to add variable constraints based on the original decomposition–coordination (dual decomposition method) MPC algorithm to solve the problem that the original method is not applicable to multivariable systems with constraints. In the first method, based on the dual decomposition method, the suboptimization problem with constraints for each subsystem forms a quadratic programming (QP) problem. The second method is the dual decomposition method based on the constrained zones, which analyzes the convergence relationship between the variables and the constraints. It proves that if the convergence factor is small enough, the solutions will eventually definitely converge to the boundaries of the constraints. Then, both proposed methods are discussed and analyzed based on their performances, concluding that the second method has superior online optimization capabilities, which are validated by Simulation 1. Further, a distributed DLMPC algorithm based on the dual decomposition of constraint zones is proposed, where the decomposition–coordinated dynamic control layer simultaneously tracks the steady-state optimized values of the controlled variable (CV) and manipulated variable (MV), which is also different from the original decomposition–coordinated dynamic control objective function, giving an optimal solution expression that added the tracking of the steady-state optimized values of the MV, and proving the effectiveness of the proposed algorithm through Simulation 2. The improved DLMPC algorithm in this paper satisfies the control goals and constraints while greatly reducing the computational complexity of the dynamic control layer, thus improving the online optimization capability of the algorithm. It is of interest and value to provide a fundamental theoretical study for the industrial implementation of distributed DLMPC.

This paper is arranged as follows: Section 1 is the Introduction. Section 2 provides an overview of DLMPC and DCMPC as a foundation for the later paper. Section 3 proposes two improved dual decomposition methods, namely, the dual decomposition method based on subsystem QP and the dual decomposition method based on the constraint zones, discussing and analyzing the performance of the two methods. Section 4 proposes a new DLMPC algorithm based on the dual decomposition method of the constraint zone. Based on the original DCMPC, which only tracks the external targets of the controlled variables, it adds the ability to track the external targets of the manipulated variable, to track the steady-state optimized value of the MV, and to give a characterization of the optimal solution under such an objective function. Section 5 simulates and validates the algorithms proposed in Section 3 and Section 4, respectively, employing the Shell heavy oil fractionation model. Section 6 is conclusions.

Partial abbreviations and notations are shown in Table 1.

## 2. Preliminary Knowledge

### 2.1. Double-Layer Model Predictive Control

**Figure 1:**
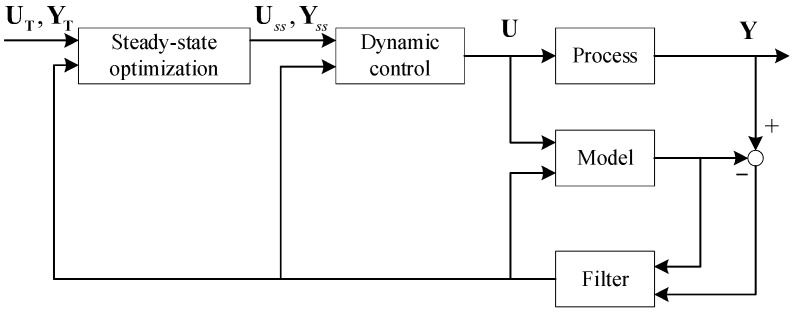
DLMPC structure.

DLMPC contains a steady-state optimization layer and dynamic control layer, and the structure is shown in Figure 1.

The steady-state optimization layer calculates the steady-state target through the steady model of plants, and the steady-state targets are tracked in the dynamic control layer. There are two modes in the steady-state optimization layer: economic optimization (EO) and target tracking (TT). The economic optimization mode characterizes the benefit maximization or energy consumption minimization as a linear programming problem with the manipulated variables, as shown in Equation (1).

(1) $$\underset{\Delta U_{\mathrm{ss}}(k)}{\min}\Xi =C^{T}*\Delta U_{\mathrm{ss}}(k)$$

where C is the economic cost coefficient. The purpose of the steady-state target tracking model is to find the optimal steady-state target based on a given RTO setpoint. Its optimization problem can be characterized as a quadratic programming problem, as shown in Equation (2).

(2) $$\underset{\Delta U_{\mathrm{ss}}(k)}{\min}\Xi ={\left\|U_{\mathrm{ss}}(k)-U_{T}\right\|}_{R_{\mathrm{ss}}}^{2}+{\left\|U_{\mathrm{ss}}(k)-Y_{T}\right\|}_{Q_{\mathrm{ss}}}^{2}+{\left\|\Delta U_{\mathrm{ss}}(k)\right\|}_{O_{\mathrm{ss}}}^{2}$$

The optimal steady-state operating point calculated by the steady-state optimization layer is used as the setpoint for tracking by the dynamic control layer, which forms the online optimization problem shown in Equation (3).

(3) $$\underset{{}_{\Delta u_{M}(k)}}{\min}J(k)={\left\|Y_{ss}(k)-{\tilde{y}}_{PM}(k)\right\|}_{Q}^{2}+{\left\|U_{ss}(k)-u_{M}(k)\right\|}_{R}^{2}+{\left\|\Delta u_{M}(k)\right\|}_{O}^{2}\;$$

where M is the control time horizon, and P is the prediction time horizon. The error power matrix and control power matrix are shown in Equation (4).

(4) $$\begin{array}{l} Q=\mathrm{block}-\mathrm{diag}(Q_{1},\cdots ,Q_{p}),Q_{i}=\mathrm{diag}[q_{i}(1),\cdots ,q_{i}(P)]\\ R=\mathrm{block}-\mathrm{diag}(R_{1},\cdots ,R_{m}),R_{j}=\mathrm{diag}[r_{j}(1),\cdots ,r_{j}(M)]\\ O=\mathrm{block}-\mathrm{diag}(O_{1},\cdots ,O_{m}),O_{j}=\mathrm{diag}[o_{j}(1),\cdots ,o_{j}(M)] \end{array}$$

where block−diag means block diagonal matrix and diag means diagonal matrix.

The dynamic prediction model A (consisting of p×m block of matrices P×M) is used to calculate the prediction value ${\tilde{y}}_{PM}(k)$ at time k as shown in Equation (5).

(5) $${\tilde{y}}_{PM}(k)={\tilde{y}}_{P0}(k)+A\Delta u_{M}(k)$$

where ${\tilde{y}}_{P0}(k)$ is the initial predicted value, consisting of the predicted value at time k−1 and the prediction error. Using the optimal solution $\Delta u_{M}^{*}(k)$ of Equation (3) to calculate the manipulated variables at time k is shown in Equation (6).

(6) $$u_{M}(k)=u_{M}(k-1)+\Delta u_{M}^{*}(k)$$

### 2.2. Decomposed–Coordinated MPC

The online optimization problem for an unconstrained multi-input multi-output (MIMO) system is shown in Equation (7).

(7) $$\begin{array}{l} \underset{{}_{\Delta u_{M}(k)}}{\min}J(k)={\left\|w(k)-{\tilde{y}}_{PM}(k)\right\|}_{Q}^{2}+{\left\|\Delta u_{M}(k)\right\|}_{R}^{2}\\ \begin{array}{cc} s.t.\; & {\tilde{y}}_{PM}(k)={\tilde{y}}_{P0}(k)+A\Delta u_{M}(k) \end{array} \end{array}$$

where we suppose the system with m inputs and m outputs and decompose the original system into m single-input single-output (SISO) subsystems. Due to the linear additivity among the subsystems, the original online optimization problem equals the sum of the predictive control sub-optimization problems, as shown in Equation (8).

(8) $$\begin{array}{l} \underset{{}_{\Delta u_{M}(k)}}{\min}J(k)=\sum_{i=1}^{m}\left\{{\left\|w_{i}(k)-{\tilde{y}}_{i,PM}(k)\right\|}_{Q_{i}}^{2}+{\left\|\Delta u_{i,M}(k)\right\|}_{R_{i}}^{2}\right\}\\ \;\begin{array}{cc} s.t. & \;{\tilde{y}}_{i,PM}(k)={\tilde{y}}_{i,P0}(k)+\sum_{j=1}^{m}A_{ij}\Delta u_{j,M}(k),\;i=1,\ldots ,m \end{array} \end{array}$$

Decomposed–Coordinated MPC originated from large-system control theory [34], a method that regards the association constraint ${\tilde{y}}_{PM}(k)$ in a coupled system as an independent variable with the same position as $\Delta u_{M}(k)$. Introducing the coordination factor λ calculates $\Delta u_{i,M}(k)$ and ${\tilde{y}}_{i,PM}(k)$ separately for each subsystem. By iterating and updating the coordination factor, so that ${\tilde{y}}_{PM}(k)$ and $\Delta u_{M}(k)$ are associated in equilibrium, the optimal solution $\Delta u_{M}(k)$ is obtained as equivalent to the original problem.

Firstly, by introducing Lagrange multipliers λ, the Lagrange function of Equation (8) is formed, as shown in Equation (9).

(9) $$L\left(\Delta u_{M}(k),{\tilde{y}}_{PM}(k),\lambda (k)\right)≜J(k)+\sum_{i=1}^{m}\lambda_{i}^{T}(k)\left({\tilde{y}}_{i,PM}(k)-{\tilde{y}}_{i,P0}(k)-\sum_{j=1}^{m}A_{ij}\Delta u_{j,M}(k)\right)$$

where $\lambda (k)={\left[\begin{array}{lll} \lambda_{1}^{T}(k) & \cdots & \lambda_{m}^{T}(k) \end{array}\right]}^{T}$ and $\lambda_{i}^{T}(k)=\left[\begin{array}{lll} \lambda_{i}(k+1) & \cdots & \lambda_{i}(k+P) \end{array}\right]$, i=1,…,m. Then, the dual problem is solved, as shown in Equation (10), and the obtained unconstrained optimal solution is that of the original optimization problem.

(10) $$\underset{{}_{\lambda (k)}}{\max}\underset{{}_{\Delta u_{M}(k),{\tilde{y}}_{PM}(k)}}{\min}L\left(\Delta u_{M}(k),{\tilde{y}}_{PM}(k),\lambda (k)\right)$$

The whole problem is decomposed into m sub-optimization problems by solving the sub-optimization problem for $\Delta u_{i,M}(k)$ and ${\tilde{y}}_{i,PM}(k)$, respectively, and then updating the coordination factor λ(k) and iterating until the stopping condition. The procedure can be depicted as a two-stage optimization algorithm.

In the first stage, the Lagrange function of the original problem is minimized for a given coordination factor $\hat{\lambda }(k)$ (Equation (9)), which can be expressed as the sum of the Lagrange functions of multiple sub-optimization problems, as shown in Equation (11).

(11) $$\underset{{}_{\Delta u_{M}(k),{\tilde{y}}_{PM}(k)}}{\min}L\left(\Delta u_{M}(k),{\tilde{y}}_{PM}(k),\hat{\lambda }(k)\right)=\sum_{i=1}^{m}L_{i}\left(\Delta u_{i,M}(k),{\tilde{y}}_{i,PM}(k),\hat{\lambda }(k)\right)$$

where $L_{i}$ is shown in Equation (12).

(12) $$\begin{array}{l} L_{i}\left(\Delta u_{i,M}(k),{\tilde{y}}_{i,PM}(k),\hat{\lambda }(k)\right)\\ ≜{\left\|w_{i}(k)-{\tilde{y}}_{i,PM}(k)\right\|}_{Q_{i}}^{2}+{\left\|\Delta u_{i,M}(k)\right\|}_{R_{i}}^{2}\\ +{\hat{\lambda }}_{i}^{T}(k)({\tilde{y}}_{i,PM}(k)-{\tilde{y}}_{i,P0}(k))-\sum_{j=1}^{m}\left({\hat{\lambda }}_{j}^{T}(k)A_{ji}\right)\Delta u_{i,M}(k) \end{array}$$

According to the extreme value necessary condition, we can obtain ${\tilde{y}}_{i,PM}^{*}(k)$ and $\Delta u_{i,M}^{*}(k)$, as shown in Equations (13) and (14).

(13) $${\tilde{y}}_{i,PM}^{*}(k)=w_{i}(k)-0.5Q_{i}^{-1}{\hat{\lambda }}_{i}(k)$$

(14) $$\Delta u_{i,M}^{*}(k)=0.5R_{i}^{-1}\sum_{j=1}^{m}\left(A_{ji}^{T}{\hat{\lambda }}_{j}(k)\right)$$

In the second stage, the coordination factor $\hat{\lambda }(k)$ is updated according to the solution of $\underset{\hat{\lambda }(k)}{\max}\varphi (\hat{\lambda }(k))$ using ${\tilde{y}}_{i,PM}^{*}(k)$ and $\Delta u_{i,M}^{*}(k)$, where $\varphi (\hat{\lambda }(k))$ is shown in Equation (15).

(15) $$\varphi (\hat{\lambda }(k))≜L\left(\Delta u_{M}^{*}(k),{\tilde{y}}_{PM}^{*}(k),\hat{\lambda }(k)\right)=\sum_{i=1}^{m}L_{i}\left(\Delta u_{i,M}^{*}(k),{\tilde{y}}_{i,PM}^{*}(k),\hat{\lambda }(k)\right)$$

$\hat{\lambda }(k)$ is modified by the gradient algorithm, as shown in Equation (16), where l is the number of iterations and α(k) is the iteration step size.

(16) $$\{\begin{array}{l} {\hat{\lambda }}_{i}^{l+1}(k)={\hat{\lambda }}_{i}^{l}(k)+\alpha (k)\nabla \gamma ({\hat{\lambda }}_{i}^{l})\\ \nabla \gamma ({\hat{\lambda }}_{i}^{l})≜\frac{\partial \varphi (\hat{\lambda }(k))}{\partial {\hat{\lambda }}_{i}^{l}(k)}={\tilde{y}}_{i,PM}^{l}(k)-{\tilde{y}}_{i,P0}(k)-\sum_{j=1}^{m}A_{ij}\Delta u_{j,M}^{l}(k) \end{array}$$

Once the difference between the two neighboring iterations’ coordination factors is sufficiently small, it means that ${\tilde{y}}_{PM}(k)$ and $\Delta u_{M}(k)$ have reached a state of associative equilibrium. The stopping condition of the iteration is shown in Equation (17).

(17) $$\left\|{\hat{\lambda }}_{i}^{l+1}(k)-{\hat{\lambda }}_{i}^{l}(k)\right\|<\varepsilon ,\;i=1,\ldots ,m$$

The relation between the solution of the decomposition–coordination method and the optimal solution of the original problem has been shown in [2], where as long as the iterative process converges, the final solution $\Delta u_{M}^{*}(k)$ is the optimal solution of the centralized solution of the original problem, as demonstrated in detail in [2].

## 3. Constrained Decomposition–Coordination Strategy

The original DCMPC is oriented towards multivariable systems with no constraints (only one equation constraint). However, most systems have constraint requirements on the controlled and manipulated variables. This paper proposes an improved DCMPC method for constrained multivariable systems, incorporating the simple handling of inequality constraints to the original method. The improved DCMPC can meet the control requirements of systems with inequality constraints.

### 3.1. Problem Description

Suppose a constrained MIMO system, with m inputs and m outputs, has additional constraints on the CV, MV, and MV increments compared to the unconstrained system. The online optimization problem formed using a centralized optimization approach is shown in Equation (18).

(18) $$\begin{array}{l} \begin{array}{l} \underset{\Delta u_{M}(k)}{\min}J(k)={\left\|w(k)-{\tilde{y}}_{PM}(k)\right\|}_{Q}^{2}+{\left\|\Delta u_{M}(k)\right\|}_{R}^{2} \end{array}\\ \begin{array}{ll} s.t. & {\tilde{y}}_{PM}(k)={\tilde{y}}_{P0}(k)+A\Delta u_{M}(k) \end{array}\\ \begin{array}{ll} & \;\;\;\;\;\;\;u_{M}(k)=u_{M}(k-1)+\Delta u_{M}(k) \end{array}\\ \begin{array}{ll} & \;\;\;\;\;\;\;\underline{Y}\leq {\tilde{y}}_{PM}(k)\leq \bar{Y} \end{array}\\ \begin{array}{ll} & \;\;\;\;\;\;\;\underline{U}\leq u_{M}(k)\leq \bar{U} \end{array}\\ \begin{array}{ll} & \;\;\;\;\;\;\;\Delta \underline{U}\leq \Delta u_{M}(k)\leq \Delta \bar{U} \end{array} \end{array}$$

Decomposing the original system into m SISO subsystems with linear additivity between the subsystems, the original centralized optimization problem is rewritten, as shown in Equation (19).

(19) $$\begin{array}{l} \underset{{}_{\Delta u_{M}(k)}}{\min}J(k)=\sum_{i=1}^{m}\left\{{\left\|w_{i}(k)-{\tilde{y}}_{i,PM}(k)\right\|}_{Q_{i}}^{2}+{\left\|\Delta u_{i,M}(k)\right\|}_{R_{i}}^{2}\right\}\\ \begin{array}{cc} \;\begin{array}{cc} s.t. & \end{array} & {\tilde{y}}_{i,PM}(k)={\tilde{y}}_{i,P0}(k)+\sum_{j=1}^{m}A_{ij}\Delta u_{j,M}(k),\;i=1,\ldots ,m\\ & u_{i,M}(k)=u_{i,M}(k-1)+\Delta u_{i,M}(k)\\ & {\underline{Y}}_{i}\leq {\tilde{y}}_{i,PM}(k)\leq {\bar{Y}}_{i}\\ & {\underline{U}}_{i}\leq u_{i,M}(k)\leq {\bar{U}}_{i}\\ & \Delta {\underline{U}}_{i}\leq \Delta u_{i,M}(k)\leq \Delta {\bar{U}}_{i} \end{array} \end{array}$$

### 3.2. The Dual Decomposition Method Based on Subsystem QP

The first improved dual decomposition method proposed in this paper for constrained MIMO distributed systems introduces CV, MV, and MV incremental constraints into the solving process of each distributed system to form the subsystem QP problem.

The equation constraint that contains the association of ${\tilde{y}}_{i,PM}(k)$ and $\Delta u_{i,M}(k)$ is first introduced into the objective function via a Lagrange multiplier to form the dual problem for m subsystems, as shown in Equation (20).

(20) $$\begin{array}{l} \begin{array}{cc} \min & L_{i}(\Delta u_{i,M}(k),{\tilde{y}}_{i,PM}(k),\lambda ) \end{array}\\ \begin{array}{cc} s.t. & u_{i,M}(k)=u_{i,M}(k-1)+\Delta u_{i,M}(k)\\ & {\underline{Y}}_{i}\leq {\tilde{y}}_{i,PM}(k)\leq {\bar{Y}}_{i}\\ & {\underline{U}}_{i}\leq u_{i,M}(k)\leq {\bar{U}}_{i}\\ & \Delta {\underline{U}}_{i}\leq \Delta u_{i,M}(k)\leq \Delta {\bar{U}}_{i} \end{array} \end{array}$$

Therefore, the association of ${\tilde{y}}_{i,PM}(k)$ and $\Delta u_{i,M}(k)$ in the constraints no longer remains. The constraints on ${\tilde{y}}_{i,PM}(k)$ and $\Delta u_{i,M}(k)$ are already independently separable, so the optimization problem of Equation (20) is further decomposed into the QP problems corresponding to ${\tilde{y}}_{i,PM}(k)$ and $\Delta u_{i,M}(k)$, as shown in Equations (21) and (22).

(21) $$\begin{array}{l} \begin{array}{cc} \underset{{\tilde{y}}_{i,PM}(k)}{\min} & {\left\|w_{i}(k)-{\tilde{y}}_{i,PM}(k)\right\|}_{Q_{i}}^{2}+ \end{array}{\hat{\lambda }}_{i}^{T}(k)({\tilde{y}}_{i,PM}(k)-{\tilde{y}}_{i,P0}(k))\\ \begin{array}{cc} s.t. & {\underline{Y}}_{i}\leq {\tilde{y}}_{i,PM}(k)\leq {\bar{Y}}_{i} \end{array} \end{array}$$

(22) $$\begin{array}{l} \begin{array}{cc} \underset{\Delta u_{i,M}(k)}{\min} & {\left\|\Delta u_{i,M}(k)\right\|}_{R_{i}}^{2} \end{array}-\sum_{j=1}^{m}\left({\hat{\lambda }}_{j}^{T}(k)A_{ji}\right)\Delta u_{i,M}(k)\\ \begin{array}{cc} s.t. & u_{i,M}(k)=u_{i,M}(k-1)+\Delta u_{i,M}(k)\\ & {\underline{U}}_{i}\leq u_{i,M}(k)\leq {\bar{U}}_{i}\\ & \Delta {\underline{U}}_{i}\leq \Delta u_{i,M}(k)\leq \Delta {\bar{U}}_{i} \end{array} \end{array}$$

Using the QP solution method, ${\tilde{y}}_{i,PM}(k)$ and $\Delta u_{i,M}(k)$ can be obtained and brought into Equation (16) to update the coordination factor λ. The remaining steps are the same as the decomposition–coordination method and will not be repeated here.

### 3.3. The Dual Decomposition Method Based on the Subsystem Constrained Zone

The second improved dual decomposition method proposed in this paper for constrained MIMO distributed systems introduces CV, MV, and MV incremental constraints into each distributed system after the solution has been solved, and the zones formed by the constraints limit the solution.

Firstly, the problem is dealt with as an optimization problem with equality constraints, equivalent to Equation (9). Then, the steps of forming the Lagrange function are the same as the original method, as shown in Equations (10)–(12). In solving the optimal of the sub-problem stage, the proposed strategy adds the inequality constraints after obtaining ${\tilde{y}}_{i,PM}^{*}(k)$ and $\Delta u_{i,M}^{*}(k)$, as shown in Equations (23) and (24).

(23) $$\begin{array}{l} K\Delta u_{i,M}^{*}(k)={\mathrm{Sat}}_{1}[0.5R_{i}^{-1}\sum_{j=1}^{m}A_{ji}^{T}{\hat{\lambda }}_{j}(k)]\\ {\mathrm{Sat}}_{1}(\alpha )=\{\begin{array}{cc} {\bar{U}}_{i,M}-u_{i,M}(k-1) & \alpha >{\bar{U}}_{i,M}\\ \alpha & {\underline{U}}_{i,M}\leq \alpha \leq {\bar{U}}_{i,M}\\ {\underline{U}}_{i,M}-u_{i,M}(k-1) & \alpha <{\underline{U}}_{i,M} \end{array} \end{array}$$

(24) $$\begin{array}{l} {\tilde{y}}_{i,PM}^{*}(k)={\mathrm{Sat}}_{2}[w_{i}(k)-0.5Q_{i}^{-1}{\hat{\lambda }}_{j}(k)]\\ {\mathrm{Sat}}_{2}(\beta )=\{\begin{array}{cc} {\bar{Y}}_{i,PM} & \beta >{\bar{Y}}_{i,PM}\\ \beta & {\underline{Y}}_{i,PM}\leq \beta \leq {\bar{Y}}_{i,PM}\\ {\underline{Y}}_{i,PM} & \beta <{\underline{Y}}_{i,PM} \end{array} \end{array}$$

where K is the lower-unit triangular matrix of M×M. The above constraint limits are only for the case of containing ${\underline{U}}_{i}\leq u_{i,M}(k)\leq {\bar{U}}_{i}$, when simultaneously containing increments of $\Delta u_{i,M}(k)$ are considered, as shown in Equation (25).

(25) $$\Delta u_{i,M}^{*}(k)=\max(\min(\Delta {\underline{U}}_{i,M}{,K}^{-1}{\mathrm{Sat}}_{1}[0.5R_{i}^{-1}\sum_{j=1}^{m}A_{ji}^{T}{\hat{\lambda }}_{j}(k)]),\Delta {\bar{U}}_{i,M})$$

In the second stage, updating the coordination factor is the same as the original method based on calculated ${\tilde{y}}_{i,PM}^{*}(k)$ and $\Delta u_{i,M}^{*}(k)$, as shown in Equations (15)–(17). Equations (23) and (25) indicate that the manipulated variables must exist within the constraints. Whether Equation (25) can make controlled variables exist within the constraints needs further analysis.

Suppose $\beta >{\bar{Y}}_{i,PM}$ exists when ${\tilde{y}}_{i,PM}^{*}(k)$ is calculated iteratively in l times, then ${\tilde{y}}_{i,PM}^{*}(k)={\bar{Y}}_{i,PM}$ exists. According to Equation (17), when the iteration stop condition is satisfied, it approximately means $\nabla \gamma ({\hat{\lambda }}_{i}^{l})=0,i=1,\ldots ,m$, as shown in Equation (26).

(26) $${\tilde{y}}_{i,PM}^{*}(k)-{\tilde{y}}_{i,P0}(k)-\sum_{j=1}^{m}A_{ij}\Delta u_{j,M}^{*}(k)=0,i=1,\ldots ,m$$

When ${\tilde{y}}_{i,PM}^{*}(k)$ surpasses the upper limit of the constraint, then ${\tilde{y}}_{i,PM}^{*}(k)={\bar{Y}}_{i,PM}$ holds through the constraint zone, and Equation (26) is rewritten as Equation (27).

(27) $${\bar{Y}}_{i,PM}-{\tilde{y}}_{i,P0}(k)-\sum_{j=1}^{m}A_{ij}\Delta u_{j,M}^{*}(k)=0,i=1,\ldots ,m$$

When we substitute the equality constraints of Equations (9) and (14) into Equation (27), we obtain that seen in Equation (28).

(28) $${\bar{Y}}_{i,PM}-\underset{{\tilde{y}}_{i,PM}(k)}{\underbrace{\left({\tilde{y}}_{i,P0}(k)+\sum_{j=1}^{m}A_{ij}0.5R_{i}^{-1}\sum_{j=1}^{m}\left(A_{ji}^{T}{\hat{\lambda }}_{j}(k)\right)\right)}}=0,i=1,\ldots ,m$$

We can notice that the sum in parentheses in Equation (28) is the predicted value of the controlled variable in the future P time horizon generated by the control law $\Delta u_{M}(k)$. As long as the predetermined ε is a small-enough positive number, and the coordination factor is continuously updated to the iteration stop condition, it can be understood from Equation (17) that ${\bar{Y}}_{i,PM}$ and $\Delta u_{M}(k)$ can reach an associated equilibrium state. In the continuous iterative process, the result ${\tilde{y}}_{i,PM}(k)$ generated by the control law $\Delta u_{M}(k)$ will eventually converge to ${\bar{Y}}_{i,PM}$. Similarly, $\beta <{\underline{Y}}_{i,PM}$ converges to ${\underline{Y}}_{i,PM}$ when $\beta <{\underline{Y}}_{i,PM}$. It is proved that the added inequality constraint strategy is also effective for the controlled variables.

### 3.4. Performance Comparison of the Proposed Algorithms

This paper proposes two improved methods based on the decomposition–coordination method for constrained distributed MPC systems. As demonstrated by the simulations in Section 5.1, both methods can satisfy the demand for constraints. However, the first method has greater computational effort than the second.

The first method introduces the equation constraint containing the association of ${\tilde{y}}_{i,PM}(k)$ and $\Delta u_{i,M}(k)$ into the dual problem $L_{i}$ by Lagrange multipliers, so that $L_{i}$ is only related to ${\tilde{y}}_{i,PM}(k)$ and $\Delta u_{i,M}(k)$, rather than to the other subsystem variables, thus decomposing the coupling between the subsystems. At the same time, ${\tilde{y}}_{i,PM}(k)$ and $\Delta u_{i,M}(k)$ are independently separable in the constraints, so that $L_{i}$ is further decomposed into the two QP problems ${\tilde{y}}_{i,PM}(k)$ and $\Delta u_{i,M}(k)$, which is equivalent to dividing the original centralized optimization problem into sub-optimization problems. For QP calculations containing a large matrix, this method can significantly reduce the computational scale. However, as suboptimization problems become more extensive, the computation time is not advantageous for some small and medium-sized computation scales. The second method is based on the original decomposition–coordination method, without adding the QP calculation, which is only through the logical determination of MV and CV constraints, so its computation time has a significant advantage compared with the first method. Compared with the centralized optimization method, the second method uses distributed parallel online iterative operations to avoid the high-dimensional matrix inverse calculation, so it has more superiority in computing control laws. The specific algorithm comparison analysis is detailed in Section 5.1.

## 4. PDLMPC Algorithm

**Figure 2:**
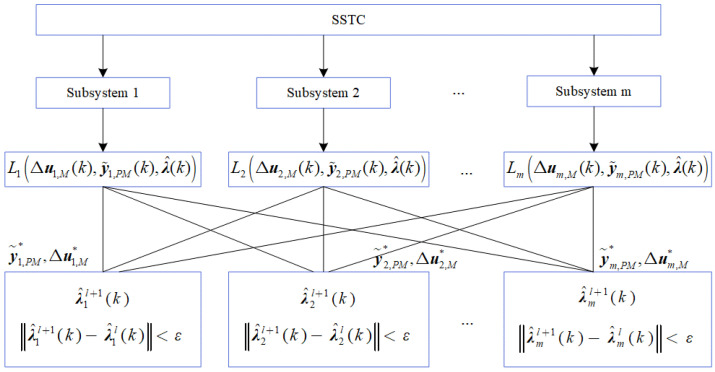
PDLMPC structure.

This paper proposes two improved dual decomposition methods for constrained multivariate systems. After the above discussion and analysis and subsequent validation, the online optimization performance of the dual decomposition method based on the constrained zone is more advantageous, so an improved PDLMPC method is introduced into the DLMPC architecture. In this method, the steady-state optimization layer adopts the centralized optimization method, and the dynamic control layer adopts the constrained decomposition–coordination method. The structure is shown in Figure 2.

Different from the matrix dimension of the dynamic model, the steady-state model has a smaller one, and it is a one-step optimization. The prediction and control horizon are both one, so the centralized optimization will not generate a large computational burden. The centralized optimization problem for the steady-state layer of a constrained multivariable system is shown in Equation (29).

(29) $$\begin{array}{l} \underset{\Delta U_{\mathrm{ss}}(k)}{\min}\Xi ={\left\|U_{\mathrm{ss}}(k)-U_{T}\right\|}_{R_{\mathrm{ss}}}^{2}+{\left\|Y_{\mathrm{ss}}(k)-Y_{T}\right\|}_{Q_{\mathrm{ss}}}^{2}+{\left\|\Delta U_{\mathrm{ss}}(k)\right\|}_{O_{\mathrm{ss}}}^{2}\\ \begin{array}{cc} s.t.\; & U_{\mathrm{ss}}(k)=U_{\mathrm{ss}}(k-1)+\Delta U_{\mathrm{ss}}(k) \end{array}\\ \begin{array}{cc} & \;\;\;\;\;\;\;Y_{\mathrm{ss}}(k)=Y_{\mathrm{ss}}(k-1)+G_{\mathrm{ss}}\Delta U_{\mathrm{ss}}(k)+e(k) \end{array}\\ \begin{array}{cc} & \;\;\;\;\;\;\;{\underline{Y}}_{\mathrm{ss}}\leq Y_{\mathrm{ss}}(k)\leq {\bar{Y}}_{\mathrm{ss}} \end{array}\\ \begin{array}{cc} & \;\;\;\;\;\;\;{\underline{U}}_{\mathrm{ss}}\leq U_{\mathrm{ss}}(k)\leq {\bar{U}}_{\mathrm{ss}} \end{array} \end{array}$$

where e(k) is the prediction error passed by the dynamic control layer. Solving Equation (24) to obtain the steady-state target values $Y_{ss}(k)$ and $U_{ss}(k)$, the online optimization problem of the dynamic control layer at time k is shown in Equation (30).

(30) $$\begin{array}{l} \underset{\Delta u_{M}(k)}{\min}J(k)={\left\|Y_{ss}(k)-{\tilde{y}}_{PM}(k)\right\|}_{Q}^{2}+{\left\|U_{ss}(k)-u_{M}(k)\right\|}_{O}^{2}+{\left\|\Delta u_{M}(k)\right\|}_{R}^{2}\\ \begin{array}{ll} s.t.\; & {\tilde{y}}_{PM}(k)={\tilde{y}}_{P0}(k)+A\Delta u_{M}(k) \end{array}\\ \begin{array}{ll} & \;\;\;\;\;\;\;u_{M}(k)=u_{M}(k-1)+\Delta u_{M}(k) \end{array}\\ \begin{array}{cc} & \;\;\;\;\;\;\;\underline{Y}\leq {\tilde{y}}_{PM}(k)\leq \bar{Y} \end{array}\\ \begin{array}{cc} & \;\;\;\;\;\;\;\underline{U}\leq u_{M}(k)\leq \bar{U} \end{array}\\ \begin{array}{cc} & \;\;\;\;\;\;\;\Delta \underline{U}\leq \Delta u_{M}(k)\leq \Delta \bar{U} \end{array} \end{array}$$

Firstly, the inequality constraints are ignored, and only the dual problem under equality constraints is considered, as shown in Equation (31).

(31) $$\begin{array}{l} L\left(\Delta u_{M}(k),{\tilde{y}}_{PM}(k),\hat{\lambda }(k)\right)\\ =\sum_{i=1}^{m}\left\{{\left\|Y_{i,ss}(k)-{\tilde{y}}_{i,PM}(k)\right\|}_{Q_{i}}^{2}+{\left\|U_{i,ss}(k)-u_{i,M}(k)\right\|}_{{}_{O_{i}}}^{2}+{\left\|\Delta u_{i,M}(k)\right\|}_{R_{i}}^{2}\right\}+\sum_{i=1}^{m}{\hat{\lambda }}_{i}^{T}(k)\left({\tilde{y}}_{i,PM}(k)-{\tilde{y}}_{i,P0}(k)-\sum_{j=1}^{m}A_{ij}\Delta u_{j,M}(k)\right)\\ =\sum_{i=1}^{m}\{{\left\|w_{i}(k)-{\tilde{y}}_{i,PM}(k)\right\|}_{Q_{i}}^{2}+{\left\|U_{i,ss}(k)-u_{i,M}(k)\right\|}_{{}_{O_{i}}}^{2}+{\left\|\Delta u_{i,M}(k)\right\|}_{R_{i}}^{2}+{\hat{\lambda }}_{i}^{T}(k)({\tilde{y}}_{i,PM}(k)-{\tilde{y}}_{i,P0}(k))\}-\sum_{j=1}^{m}\sum_{i=1}^{m}({\hat{\lambda }}_{i}^{T}(k)A_{ij}\Delta u_{j,M}(k))\\ =\sum_{i=1}^{m}L_{i}\left(\Delta u_{i,M}(k),{\tilde{y}}_{i,PM}(k),\hat{\lambda }(k)\right) \end{array}$$

At the first level, the dual problem $\underset{{}_{\Delta u_{M}(k),{\tilde{y}}_{PM}(k)}}{\min}L\left(\Delta u_{M}(k),{\tilde{y}}_{PM}(k),\hat{\lambda }(k)\right)$ is minimized to solve $\Delta u_{i,M}^{*}(k)$ and ${\tilde{y}}_{i,PM}^{*}(k)$, as shown in Equations (32) and (33).

(32) $$\begin{array}{l} \Delta u_{i,M}^{*}(k)=\max(\min({\underline{U}}_{i},\Delta {\mathrm{Sat}}_{1}[0.5{\left(R_{i}+O_{i}\right)}^{-1}(\sum_{j=1}^{m}A_{ji}^{T}{\hat{\lambda }}_{j}(k)+2(U_{i,ss}(k)-u_{i,M}(k-1))]),\Delta {\bar{U}}_{i})\\ {\mathrm{Sat}}_{1}(\alpha )=\{\begin{array}{cc} {\bar{U}}_{i,M}-u_{i,M}(k-1) & \alpha >{\bar{U}}_{i,M}\\ \alpha & {\underline{U}}_{i,M}\leq \alpha \leq {\bar{U}}_{i,M}\\ {\underline{U}}_{i,M}-u_{i,M}(k-1) & \alpha <{\underline{U}}_{i,M} \end{array} \end{array}$$

(33) $$\begin{array}{l} {\tilde{y}}_{i,PM}^{*}(k)={\mathrm{Sat}}_{2}[Y_{i,ss}(k)-0.5Q_{i}^{-1}{\hat{\lambda }}_{j}(k)]\\ {\mathrm{Sat}}_{2}(\beta )=\{\begin{array}{cc} {\bar{Y}}_{i,PM} & \beta >{\bar{Y}}_{i,PM}\\ \beta & {\underline{Y}}_{i,PM}\leq \beta \leq {\bar{Y}}_{i,PM}\\ {\underline{Y}}_{i,PM} & \beta <{\underline{Y}}_{i,PM} \end{array} \end{array}$$

The steps for updating the coordination factor in the second stage are the same as the original method. That is, Equations (15)–(17) are performed. When the iteration is stopped, the optimal manipulated variable is applied to the controlled process. The deviation between the sampling result y(k+1) at time k+1 and the predicted value ${\tilde{y}}_{N}(k+1|k)$ at time k+1 is taken as the prediction error e(k+1). The prediction error is transmitted to the steady-state optimization layer and the feedback correction module. The predicted initial value ${\tilde{y}}_{N0}(k+1)$ is obtained after feedback correction and shifting, as shown in Equation (34).

(34) $$\{\begin{array}{c} {\tilde{y}}_{\mathrm{cor}}(k+1)={\tilde{y}}_{N}(k)+he(k+1)\\ {\tilde{y}}_{N0}(k+1)=S{\tilde{y}}_{\mathrm{cor}\;}(k+1) \end{array}$$

In summary, the improved DLMPC algorithm based on the decomposition–coordination method for constrained multivariable systems is complete. The algorithm adopts the overall optimization mode in the steady-state layer, which can give a more comprehensive steady-state target value. Taking the steady-state target value as the setting point of the dynamic control layer can provide more global information for each subsystem and more reasonable tracking targets. The distributed architecture of the dynamic control layer ensures the information transmission between subsystems through decomposition and coordination. At the same time, a simple constraint method is proposed. Through theoretical analysis, it is proved that the method can ensure that MV and CV run in the constraint conditions. However, this method is also incomplete and needs specific skills and experience when setting coefficients such as weight of control, error weight, and iteration stop accuracy.

## 5. Simulation

**Figure 3:**
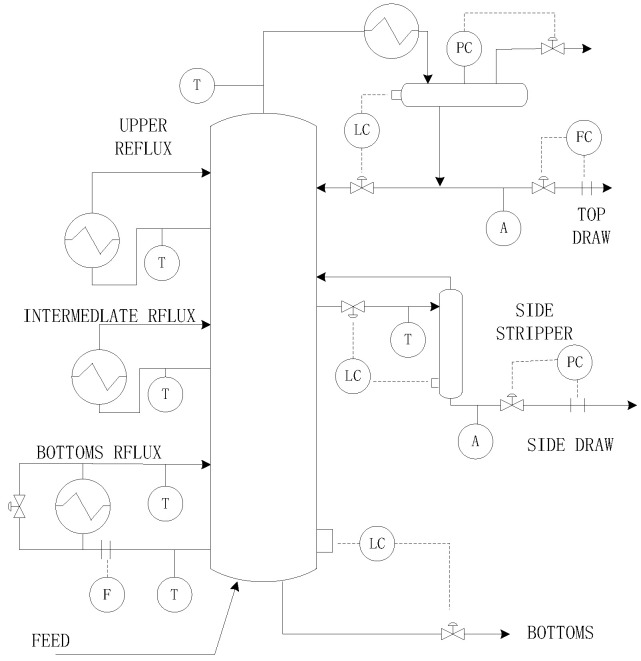
Shell heavy oil fractionation structure.

The simulation object used in this paper is the Shell heavy oil fractionation tower model, which is a typical, large, constrained, multivariable process, the structure of which is shown in Figure 3. After simplification, it can be regarded as a linear model with three inputs and three outputs, as shown in Equation (35). The controlled variables are product draw $y_{1}$, side product draw $y_{2}$, and bottom reflux temperature $y_{3}$. The manipulated variables are the top flow $u_{1}$, side flow $u_{2}$, and bottom reflux heat transmission rate $u_{3}$.

(35) $$\left[\begin{array}{c} y_{1}\\ y_{2}\\ y_{3} \end{array}\right]=\left[\begin{array}{ccc} \frac{4.05e^{-27s}}{50s+1} & \frac{1.77e^{-28s}}{60s+1} & \frac{5.88e^{-27s}}{50s+1}\\ \frac{5.39e^{-18s}}{50s+1} & \frac{5.72e^{-14s}}{60s+1} & \frac{6.90e^{-15s}}{40s+1}\\ \frac{4.38e^{-20s}}{33s+1} & \frac{4.42e^{-22s}}{44s+1} & \frac{7.20}{19s+1} \end{array}\right]\cdot \left[\begin{array}{c} u_{1}\\ u_{2}\\ u_{3} \end{array}\right]$$

### 5.1. PDD1 and PDD2 Validation of the Static Model

To verify the performance and effectiveness of the two algorithms proposed in Section 4, the static model of Shell heavy oil fractionation with both a prediction and control time horizon of 1 is employed, as shown in Equation (36). The optimization problem adopts the form of Equation (18), which tracks only the external target of the controlled variable. This optimization problem is required to satisfy the CV and MV constraints, as shown in Table 2. The comparison algorithm is the original centralized optimization method (OC), verifying that the optimal solution sought by the improved dual decomposition method based on the subsystem QP (PDD1) and the improved dual decomposition method based on the subsystem constraint zone (PDD2) is consistent with the optimal solution of the CMPC. The control goal is to achieve a given target value $Y_{T}={\left[\begin{array}{ccc} 0.9 & 0.9 & 0.9 \end{array}\right]}^{T}$ for the CV while satisfying the constraints (as shown in Table 2). The simulation step size is 100.

(36) $$\left[\begin{array}{c} y_{1}\\ y_{2}\\ y_{3} \end{array}\right]=\left[\begin{array}{ccc} 4.05 & 1.77 & 5.88\\ 5.39 & 5.72 & 6.90\\ 4.38 & 4.42 & 7.2 \end{array}\right]\cdot \left[\begin{array}{c} u_{1}\\ u_{2}\\ u_{3} \end{array}\right]$$

**Figure 4:**
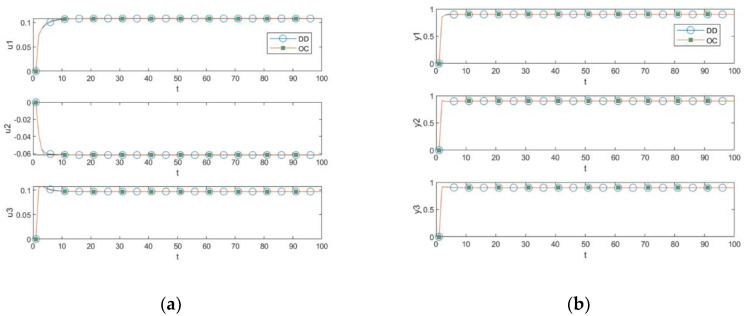
Comparisons for the unconstrained case. (**a**) MV comparison; (**b**) CV comparison.

Simulations are first performed in the unconstrained case to ensure that the constraints in Table 2 are positive, comparing the algorithms to the original decomposition–coordination method (DD) and OC. The simulation results are shown in Figure 4. The optimal solution trajectories of OC and DD can be seen to overlap almost exactly, with $u_{1}$ and $u_{3}$ in Figure 4a both exceeding the constraint upper bound of 0.1, proving that the added constraint is positive.

**Figure 5:**
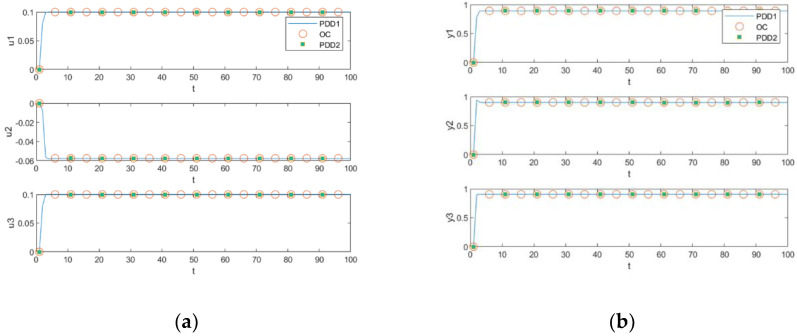
Comparisons for the constrained case. (**a**) MV comparison; (**b**) CV comparison.

Comparing the centralized optimization method with constraints, the PDD1 and PDD2 are proposed in this paper. The results show that the three algorithms’ optimal solutions are consistent in the presence of constraints, as shown in Figure 5. Comparing the optimal solution trajectory of $u_{1}$ and $u_{3}$ in Figure 4a and Figure 5a, $u_{1}$ and $u_{3}$ are obviously constrained by the constraints. In Figure 5b, the CVs of the proposed algorithms meet the external target values required for control, and the trajectories of the CVs can track the EVs without zero deviation. The constraints on the CVs show that the CVs have residual degrees of freedom, which means that the CVs track without zero deviation not due to the positive constraints that passively form the control trajectories, but rather due to the naturally occurring control trajectory formed in response to the control law.

As for the algorithm running time, PDD1 took about 14.68 s, OC took about 0.522 s, and PDD2 took about 0.0259 s. We noticed that PDD1 takes the longest time and PDD2 has an obvious advantage in reducing the amount of computation and shortening the running time, which verifies the conjecture in Section 3.4.

In summary, Simulation 1 demonstrates that the constrained decomposition–coordination method proposed in Section 4 can ensure that the variables are constrained. At the same time, the control goal can be met under positive constraints. The resulting optimal solution trajectory is consistent with the constrained CMPC algorithm, verifying the effectiveness of the proposed algorithm and laying the foundation for the introduction of DLMPC.

The above simulation results show the effectiveness of the two improved dual decomposition methods proposed in this paper. Both methods can ensure that the variables are constrained and the control goal can be achieved under positive constraints. The optimal solution trajectory is consistent with the constrained OC algorithm. At the same time, the calculation time of the three algorithms is compared. PDD2 takes the shortest time and shows excellent online optimization performance.

### 5.2. PDLMPC Algorithm Verification Based on PDD2

Although PDD1 achieves results consistent with OC, it takes longer. It is not advantageous for small- to medium-sized solutions, so we use a modified double-layer model predictive control algorithm (PDLMPC) based on PDD2 for subsequent simulation validation and a comparison algorithm using the original double-layer model predictive control algorithm (ODLMPC) with centralized optimization, using the Shell heavy oil fractionation model as shown in Equation (35). The steady-state optimization layer is required to track the external value for a given MV and CV and constitutes the steady-state optimization problem, as shown in Equation (18). The dynamic control layer tracks the steady-state targets $Y_{\mathrm{ss}}$, as shown in Equation (25). The control goal is to bring the CV to the given target value $Y_{T}={\left[\begin{array}{ccc} 0.5 & 0.5 & 0.5 \end{array}\right]}^{T}$ while satisfying the constraints in Table 3.

**Figure 6:**
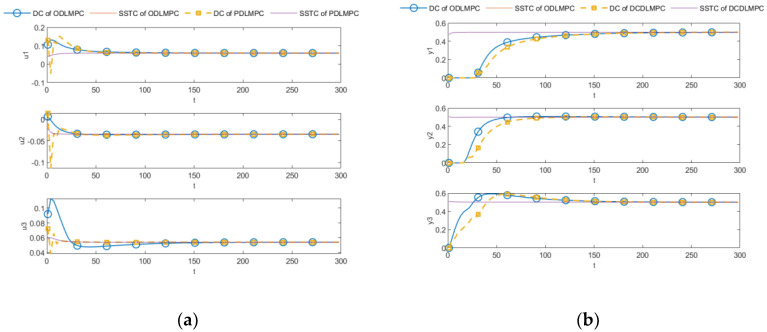
Comparisons of DCDLMPC and ODLMPC. (**a**) MV comparison; (**b**) CV comparison.

The ODLMPC and DCDLMPC (centralized steady-state optimization for the upper layer and decomposed coordinated control for the lower layer) algorithms were first run without constraints to prove that the constraints in Table 3 are positive, and the simulation results are shown in Figure 6. The MVs of both ODLMPC and DCDLMPC algorithms exceeded the constraint limits in the unconstrained case, proving that the constraints in Table 3 are positive constraints. Ensuring that the controlled variable eventually reaches the control target is not the constraint forcing the trajectory to form so that the constraint on the controlled variable is greater than the control target value. It is worth noting that in Figure 6 the results of the two control algorithms are inconsistent, with the control results of DCDLMPC being more advantageous. This situation is because the control and error weight parameters of DLMPC are not set correctly.

**Figure 7:**
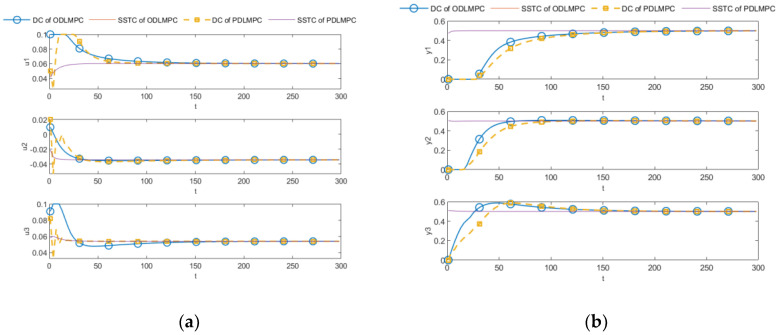
Comparisons of PDLMPC and ODLMPC. (**a**) MV comparison; (**b**) CV comparison.

Results from the ODLMPC and the PDLMPC algorithms presented in Section 5, running with constraints, are shown in Figure 7. Figure 7a clearly shows that the MV under the PDLMPC algorithm is restricted by the same constraints as the ODLMPC and that both the CV and MV are able to track the steady-state optimization trajectory given by the SSTC layer to realize the control goal. The average time taken for five runs of the DLMPC algorithm was 4.87 s, and the average time taken for five runs of the PDLMPC algorithm was 2.58 s, where the controllers of the three subsystems were not run in parallel but in series, which would have been smaller if they had been run in parallel. The PDLMPC algorithm reduces the computational complexity with less computational burden by giving up some of the tracking performance, and this sub-optimal result is acceptable.

## 6. Conclusions

This paper proposes an improved distributed DLMPC approach facing a complex class of constrained systems in modern, extensive, industrial processes. Firstly, two methods for adding constraints are proposed based on the decomposition–coordination MPC. The first improved dual decomposition method introduces the constraints of MV and CV into solving the distributed subsystem, forming the QP problem with the dual function of the subsystem to solve it. The second is a dual decomposition method based on constrained zones, which introduces the constraints of MV and CV into the distributed subsystem after solving ${\tilde{y}}_{i,PM}^{*}(k)$ and $\Delta u_{i,M}^{*}(k)$. Furthermore, the convergence of the method is analyzed: as long as the parameters are set appropriately, the CV will eventually converge to the constraint boundary. The online optimization capabilities of the two proposed methods are discussed and compared, concluding that the second proposed method, the pairwise decomposition method based on constraint zones, has superior online optimization capability. This guess is proved by comparing the two proposed methods and the centralized optimization method through simulation. Based on the above work, an improved distributed DLMPC algorithm based on the pairwise decomposition method with constrained zones is proposed. Different from the objective function in the original decomposition–coordination method, the objective function used in the dynamic control layer of the improved distributed DLMPC algorithm tracks both the steady-state optimized values of MV and CV and the dynamic control layer of the decomposition–coordination with constraints designed for this objective function, which gives the characterization of the optimal solution as well as the strategy for processing the constraints. The proposed algorithm can reduce the computational complexity while achieving the control goals. The effectiveness and rationality of the proposed algorithm are validated through simulations and compared to the simulation results of DCDLMPC without constraints. It is evident that PDLMPC can make the manipulated variables constrained. Compared with ODLMPC, PDLMPC uses less running time and the control effect is similar, so it can meet the control requirements and achieve the control goals. Of course, the PDLMPC algorithm also has shortcomings; it has specific requirements for parameter settings, and further research will be carried out in the future to optimize the parameters of this algorithm.

## Figures and Tables

**Table 1:** Main abbreviations or notations.

Abbreviations/Notations	Meaning	Notations	Meaning
CV	Controlled variable	$\underline{U}$	MV constraint lower limits
MV	Manipulated variable	$\underline{Y}$	CV constraint lower limits
U	MV	$\bar{U}$	MV constraint upper limits
Y	CV	$\bar{Y}$	MV constraint upper limits
Subscript T	Tracking targets	${\mathrm{Subscript}\;}^{ss}$	Steady-state optimization layer
Δ	Increment	${\tilde{y}}_{PM}(k)$	The output predicted value of P time domain in the future under M control law at the time k
S	Shift matrix	${\tilde{y}}_{P0}(k)$	The initial output predicted value of the future P time domains at the time k
h	Correction matrix	w(k)	The reference trajectory of the controlled variable at the time k
N	Model length	$u_{M}(k)$	M control law increments at the time k
P	Prediction time horizon	$G_{\mathrm{ss}}$	Steady-state transfer function matrix
M	Control time horizon	${\tilde{y}}_{\mathrm{cor}}$	Corrected predicted values of the controlled variables

**Table 2:** Parameter setting.

Parameter Name	Parameter Value	Parameter Name	Parameter Value
CV constraint upper limit	$\bar{Y}={\left[\begin{array}{ccc} 1 & 1 & 1 \end{array}\right]}^{T}$	CV constraint lower limit	$\underline{Y}={\left[\begin{array}{ccc} -1 & -1 & -1 \end{array}\right]}^{T}$
MV constraint upper limit	$\bar{U}={\left[\begin{array}{ccc} 0.1 & 0.1 & 0.1 \end{array}\right]}^{T}$	MV constraint lower limit	$\underline{U}={\left[\begin{array}{ccc} -0.1 & -0.1 & -0.1 \end{array}\right]}^{T}$

**Table 3:** Parameter setting.

Parameter Name	Parameter Value	Parameter Name	Parameter Value
CV constraint upper limit	$\bar{Y}={\left[\begin{array}{ccc} 1 & 1 & 1 \end{array}\right]}^{T}$	CV constraint lower limit	$\underline{Y}={\left[\begin{array}{ccc} -1 & -1 & -1 \end{array}\right]}^{T}$
MV constraint upper limit	$\bar{U}={\left[\begin{array}{ccc} 0.1 & 0.1 & 0.1 \end{array}\right]}^{T}$	MV constraint lower limit	$\underline{U}={\left[\begin{array}{ccc} -0.1 & -0.1 & -0.1 \end{array}\right]}^{T}$
MV incremental constraint lower limit	$\Delta \bar{U}={\left[\begin{array}{ccc} 0.05 & 0.05 & 0.05 \end{array}\right]}^{T}$	MV incremental constraint lower limit	$\Delta \underline{U}={\left[\begin{array}{ccc} -0.05 & -0.05 & -0.05 \end{array}\right]}^{T}$

## Data Availability

Not applicable.

## References

[1] Tatjewski P. (2008). Advanced control and on-line process optimization in multilayer structures. Annu. Rev. Control.

[2] Xi Y.G., Li D.W. (2013). Predictive Control.

[3] Rawlings J.B., Mayne D.Q. (2009). Model Predictive Control: Theory and Design.

[4] Sorensen R.C., Cutler C.R. (1998). Lp integrates economics into dynamic matrix control: Process control and information systems: A special report. Hydrocarb. Process..

[5] Brosilow C., Zhao G., Leondes C.T. (1988). A Linear Programming Approach to Constrained Multivariable Process Control. System Identification and Adaptive Control.

[6] Morshedi A.M., Cutler C.R., Skrovanek T.A. Optimal solution of dynamic matrix control with linear programing techniques (ldmc). Proceedings of the 1985 American Control Conference.

[7] Qin S., Badgwell T.A. (2003). A survey of industrial model predictive control technology. Control Eng. Pract..

[8] Zou T., Ding B.C., Zhang D. (2010). MPC: An Introducion to Indus-Trial Applications.

[9] Yang Y., Ding B. (2020). Two-layer model predictive control for chemical process model with integrating controlled variables. Can. J. Chem. Eng..

[10] Liu J., Sun H., Lu Y., Hu J., Zou T. (2021). A weighted local steady-state determination approach based on the globally optimal economic steady-states. Can. J. Chem. Eng..

[11] Liu J., Sun H., Zhang Y., Hu J., Zou T. (2022). Steady-state sequence optimization with incremental input constraints in two-layer model predictive control. ISA Trans..

[12] Keviczky T., Borrelli F., Balas G.J. (2006). Decentralized receding horizon control for large scale dynamically decoupled systems. Automatica.

[13] Alessio A., Barcelli D., Bemporad A. (2011). Decentralized model predictive control of dynamically coupled linear systems. J. Process. Control.

[14] Barcelli D., Bemporad A. (2009). Decentralized Model Predictive Control of Dynamically-Coupled Linear Systems: Tracking under Packet Loss. IFAC Proc. Vol..

[15] Christofides P.D., Scattolini R., de la Peña D.M., Liu J. (2013). Distributed model predictive control: A tutorial review and future research directions. Comput. Chem. Eng..

[16] Negenborn R., Maestre J. (2014). Distributed Model Predictive Control: An Overview and Roadmap of Future Research Opportunities. IEEE Control Syst..

[17] Camisa A., Köhler P.N., Müller M.A., Notarstefano G., Allgöwer F. (2020). A distributed optimization algorithm for Nash bargaining in multi-agent systems. IFAC-PapersOnLine.

[18] Francisco M., Mezquita Y., Revollar S., Vega P., De Paz J.F. (2019). Multi-agent distributed model predictive control with fuzzy negotiation. Expert Syst. Appl..

[19] Zhou Z., De Schutter B., Lin S., Xi Y. (2017). Two-Level Hierarchical Model-Based Predictive Control for Large-Scale Urban Traffic Networks. IEEE Trans. Control Syst. Technol..

[20] Masero E., Francisco M., Maestre J.M., Revollar S., Vega P. (2021). Hierarchical distributed model predictive control based on fuzzy negotiation. Expert Syst. Appl..

[21] Farina M., Scattolini R. (2011). Distributed non-cooperative MPC with neighbor-to-neighbor communication. IFAC Proc. Vol..

[22] Hu H., Gatsis K., Morari M., Pappas G.J. (2020). Non-Cooperative Distributed MPC with Iterative Learning. IFAC-PapersOnLine.

[23] Sun B., Tang Y., Ye L., Chen C., Zhang C., Zhong W. (2018). A Frequency Control Strategy Considering Large Scale Wind Power Cluster Integration Based on Distributed Model Predictive Control. Energies.

[24] Yame J.J., Gabsi F., Darure T., Jain T., Hamelin F., Sauer N. Optimality Condition Decomposition Approach to Distributed Model Predictive Control. Proceedings of the 2019 American Control Conference (ACC).

[25] Namba T., Takeda K., Takaba K. Dual Decomposition-Based Distributed Microgrid Managament with PV Prediction. Proceedings of the 2018 57th Annual Conference of the Society of Instrument and Control Engineers of Japan (SICE).

[26] Wakasa Y., Arakawa M., Tanaka K., Akashi T. Decentralized model predictive control via dual decomposition. Proceedings of the 2008 47th IEEE Conference on Decision and Control.

[27] Zafra-Cabeza A., Maestre J.M. (2013). A Hierarchical Distributed MPC Approach: A Practical Implementation.

[28] Nabais J.L., Negenborn R.R., Carmona-Ben´ıtez R.B., Mendon¸ca L.F., Botto M.A. (2014). Hierarchical MPC for Multiple Commodity Transportation Networks.

[29] Kozma A., Savorgnan C., Diehl M. (2013). Distributed Multiple Shooting for Large Scale Nonlinear Systems.

[30] Kang W., Li Q., Chen M., Peng C., Chen F. (2018). A two-layer distributed control method for islanded microgrids by using multi-agent systems. Zhongguo Dianji Gongcheng Xuebao/Proc. Chin. Soc. Electr. Eng..

[31] Tilli A., Garone E., Conficoni C., Cacciari M., Bosso A., Bartolini A. (2022). A two-layer distributed MPC approach to thermal control of Multiprocessor Systems-on-Chip. Control Eng. Pract..

[32] Yang K., Li L., Xue F. (2016). Real-time optimization and distributed control integration algorithm. Comput. Meas. Control..

[33] Shi Y., Zhang Z., Sun P., Xie L., Chen Q., Su H., Chen X. (2021). Two-layer structure strategy for large-scale systems integrating online adaptive constraints adjustment method and cooperative distributed DMC algorithm. Control. Eng. Pract..

[34] Da Q., He J. (1989). Large Systems Theory and Methods.

